# Statistical optimization of glucose, sodium citrate and potassium chloride supplementation for enhanced bacteriocin production by *Lactococcus lactis* MT186647 and *Lactobacillus fermentum* MT186598 isolated from fermenting *Pentaclethra macrophylla* benth

**DOI:** 10.3389/frabi.2026.1907088

**Published:** 2026-07-22

**Authors:** Chijioke Ethel Onwuakor, Anayochukwu Chibuike Ngene

**Affiliations:** Department of Microbiology, College of Natural Sciences, Michael Okpara University of Agriculture, Umudike, Abia State, Nigeria

**Keywords:** bacteriocin production, food biopreservation, lactic acid bacteria, medium optimization, response surface methodology

## Abstract

Bacteriocin production by lactic acid bacteria (LAB) is strongly conditioned by growth-medium composition, and adjusting ingredients offers a low-cost route to enhanced yield. Response surface methodology (RSM) based on a three-factor, five-level central composite design (CCD) was used to optimize supplementation of an African oil bean seed (*Pentaclethra macrophylla* Benth) extract broth with glucose, sodium citrate and potassium chloride for two LAB isolated from the seed and identified by 16S rRNA sequencing, *Lactococcus lactis* MT186647 and *Lactobacillus fermentum* MT186598. Twenty runs per organism were performed; bacteriocin activity was determined by the zone of inhibition against *Staphylococcus aureus* ATCC 19095 by agar well diffusion. For both isolates, glucose exerted a significant negative linear effect and all three supplements significant negative quadratic effects, while no two-factor interaction reached significance, indicating an additive response. The second-order models accounted for 71.85% (*L. lactis*; p = 0.060) and 73.15% (*L. fermentum*; p = 0.050) of the variation, with significant lack-of-fit; the regression was therefore only marginally significant, and the predicted R² was 0.00%, indicating no demonstrated ability to predict new runs. The desirability-based optima (6.35/0.55/1.78 g/L glucose/sodium citrate/potassium chloride for *L. lactis*; 6.44/0.55/1.59 g/L for *L. fermentum*; predicted zones 13.12 and 12.74 mm) are best interpreted as an estimated optimal region rather than a quantitatively predictive optimum. Validation under these optima combined with the previously established optimal culture conditions (1.9% NaCl, 31 °C, initial pH 6.0) produced zones of 15.22 mm (*L. lactis*) and 14.96 mm (*L. fermentum*), exceeding the supplementation-only model estimates by 16.0% and 17.4% and the previously reported culture-condition-only outcomes (13.33 and 11.75 mm) by 14.2% and 27.3%. Because these differences were not tested statistically and partly reflect the fixed 1.9% NaCl (optimized separately and absent from the supplement CCD), the two optimization layers are interpreted as combining additively rather than demonstrating an established synergy. Moderate, balanced supplementation with inexpensive, food-grade additives can substantially enhance bacteriocin yield, supporting further development, subject to confirmatory multi-point validation, of *L. lactis* MT186647 and *L. fermentum* MT186598 as starter or protective cultures for the controlled fermentation of *P. macrophylla* to *Ugba*.

## 1 Introduction

### 1.1 Bacteriocins and food biopreservation

Bacteriocins are ribosomally synthesized antimicrobial peptides produced by bacteria, and those produced by lactic acid bacteria (LAB) have received particular attention as natural alternatives to chemical preservatives in foods (Darbandi et al., 2022; Ohaegbu et al., 2022; Tian et al., 2026). Their generally recognized as safe (GRAS) status, their narrow but ecologically useful activity spectrum, and increasing consumer demand for clean-label products have together fueled interest in their controlled application to food systems (John et al., 2026; Ngene et al., 2026). Although bacteriocin activity is classically directed against Gram-positive species closely related to the producer, several LAB bacteriocins, particularly under permeabilizing or stressed conditions, are also active against Gram-negative organisms (Ibejekwe et al., 2025, Ibejekwe et al., 2023).

In sub-Saharan Africa, where the cold chain is often discontinuous and chemical preservatives are not always affordable or culturally acceptable, indigenous fermented foods provide a long-established model for the *in situ* deployment of LAB-derived antimicrobials. African oil bean seed (*Pentaclethra macrophylla* Benth) is processed in south-eastern Nigeria into *Ugba*, a protein-rich condiment whose early-stage microbial succession involves LAB before being dominated by *Bacillus* species (Onwuakor et al., 2020 and Onwuakor et al., 2021). LAB isolated from this fermentation are therefore matched, by their ecological origin, to the substrate in which any future starter culture would be deployed.

### 1.2 Medium composition as a determinant of bacteriocin yield

Bacteriocin biosynthesis in LAB is primary-metabolite-like in its kinetics, with most production occurring in close association with cellular growth (Abbasiliasi et al., 2017; Chapot-Chartier and Kulakauskas, 2014). Yield is therefore subject to the same constraints as biomass formation, including the availability and balance of carbon, nitrogen, phosphorus and trace elements, the pH and buffering capacity of the medium, and the ionic environment. Carbohydrate concentration influences bacteriocin output both as a substrate for biosynthesis and through carbon catabolite repression at high levels (Castro et al., 2011; Sidooski et al., 2019). Organic anions and salts such as citrate and potassium chloride contribute to pH buffering, osmotic balance and the transport functions of LAB cells, and have been shown to modulate bacteriocin production in a strain-dependent manner (Hossain, 2022; Zhang et al., 2026).

Because bacteriocin yield depends on several medium variables simultaneously, and because their effects can be non-linear and interactive, one-factor-at-a-time (OFAT) approaches, although still common in screening studies, are at best inefficient and at worst misleading (Onwuakor et al., 2014; Yolmeh and Jafari, 2017). They cannot capture quadratic optima or factor interactions, and the optimum identified by sequential OFAT is conditional on the starting point.

### 1.3 Response surface methodology

Response surface methodology (RSM) addresses these limitations by modeling the joint linear, quadratic and interactive effects of several factors using a comparatively small number of designed experiments (Sarabia and Ortiz, 2009; Yolmeh and Jafari, 2017). Among the available designs, the central composite design (CCD) is widely used because it allows fitting of full second-order polynomial models with relatively few runs and provides good predictions across a continuous experimental region. RSM with CCD has been applied to the optimization of bacteriocin and bacteriocin-like inhibitory substance (BLIS) production in several LAB and related organisms, including *Lactobacillus* casei (Kumar et al., 2012), *Lactobacillus* brevis DF01 (Lee et al., 2012), *Pediococcus* acidilactici BA28 (Kaur et al., 2013), *Pediococcus* pentosaceus KC692718 (Suganthi and Mohanasrinivasan, 2015), *Lactobacillus* delbrueckii subsp. bulgaricus (Radha and Padmavathi, 2017), *Lactobacillus* plantarum (Le et al., 2019) and *Bacillus* licheniformis P40 (Cladera-Olivera et al., 2004), with each study reporting a model-defined optimum that improved on previous OFAT outcomes.

### 1.4 Aim of the present study

Two LAB previously isolated from fermenting *P. macrophylla*, *L. lactis* MT186647 and *L. fermentum* MT186598, have already been optimized for bacteriocin production with respect to the physical culture conditions (temperature, pH and sodium chloride concentration) in earlier publications. What had not been determined for these strains is the influence of common medium supplements (carbohydrate, organic anion buffer and potassium salt) on their bacteriocin output, or whether the supplement and culture-condition optimizations combine constructively. The present design deliberately optimized the medium supplements separately from, and subsequent to, the previously reported culture-condition optimization; we note at the outset that this sequential, two-stage strategy is less integrated than a single expanded design that would incorporate all critical factors simultaneously, a limitation we return to in Section 4. The present study therefore (i) applied an identical three-factor CCD to optimize glucose, sodium citrate and potassium chloride supplementation for each isolate; (ii) compared the response surfaces, coefficients and optima of the two strains side by side; and (iii) validated the supplementation optima in combination with the previously established optimal culture conditions, to test whether the two layers of optimization act additively or synergistically.

## 2 Materials and methods

### 2.1 Microorganisms and indicator strain

The bacteriocin-producing strains *Lactococcus lactis* MT186647 and *Lactobacillus fermentum* MT186598 (Onwuakor et al., 2020), previously isolated from fermenting African oil bean seed (*P. macrophylla*) and identified by 16S rRNA gene sequencing (sequences deposited with NCBI GenBank under the cited accession numbers), were used. Working stock cultures were maintained on de Man, Rogosa and Sharpe (MRS) agar slants at 4 °C and propagated in MRS broth at 35 °C for 24 h before each experiment. The indicator strain *Staphylococcus aureus* ATCC 19095, obtained from the Federal Institute of Industrial Research, Oshodi (FIIRO), Lagos, Nigeria, was maintained on mannitol salt agar at 37 °C and sub-cultured in nutrient broth, with long-term storage at −20 °C in 20% (v/v) glycerol.

### 2.2 Fermentation medium and bacteriocin production

Fermentations were carried out in 100 mL portions of an African oil bean seed (AOBS) extract broth, prepared by boiling, dehulling, slicing, blending and aqueous extraction of the seed essentially as described in earlier work, supplemented with glucose, sodium citrate and potassium chloride at the concentrations dictated by the CCD. Each flask was inoculated with 1% (v/v) of an overnight (24 h) MRS culture of the respective producer organism and incubated at 31 °C with an initial pH of 6.0 for 24 h. The initial pH was set to 6.0 but was not controlled during incubation, and the final pH of each run was not recorded; the implications of this for the interpretation of the glucose effect are considered in Section 4.1. After incubation, cells were removed by centrifugation at 10–000 rpm for 15 min at 4 °C. The cell-free supernatant was adjusted to pH 6.5 with 1 mol/L NaOH to exclude any contribution from residual organic acids, partially purified by ammonium sulfate precipitation and redissolution in double-distilled water, and filtered through a 0.22 µm syringe filter.

### 2.3 Antibacterial activity assay

Bacteriocin activity was determined by the agar well diffusion method (Musa et al., 2023) and is reported throughout as the zone of inhibition (ZI), in millimeters. Briefly, Mueller-Hinton agar plates were surface-inoculated with an overnight nutrient broth culture of *S. aureus* ATCC 19095 adjusted to approximately 0.5 McFarland turbidity. Wells of 6 mm diameter were cut into the seeded agar, 100 µL of the partially purified bacteriocin preparation was dispensed into each well, plates were allowed to pre-diffuse at ambient temperature for 2 h, and then incubated at 37 °C for 24 h. The ZI was recorded as the diameter of the clear inhibition zone surrounding each well, including the well, measured in two perpendicular directions and averaged. All fermentations and assays were carried out in duplicate; the coefficient of variation (CV) between duplicates did not exceed 4.5% in any run. We acknowledge that triplicate (rather than duplicate) replication is generally recommended for RSM-CCD studies to obtain a more robust estimate of pure error; the consequences of duplicate replication for the confidence of the fitted models are discussed in Section 4.5.

### 2.4 Experimental design

A three-factor, five-level (−α, −1, 0, + 1, +α; α = 1.68) face-centered CCD was employed for each producer organism, comprising 20 runs of 8 factorial, 6 axial and 6 center points. The independent variables were X_1_ = glucose, X_2_ = sodium citrate and X_3_ = potassium chloride, varied at the factorial levels 5.0-10.0 g/L, 0.3-1.0 g/L and 0.55-3.0 g/L, respectively; the axial levels were computed from these ranges. The response (Y) was the zone of inhibition against *S. aureus* ATCC 19095. The order of the 20 experimental runs was fully randomized for each organism using the run-order randomization function of the design software, so that factorial, axial and center points were interspersed; this was done to protect the analysis against systematic bias arising from any time-dependent drift in inoculum condition, incubator performance or assay reading. A second-order polynomial of the form.


$$Y=\beta_{0}+\sum_{i}\beta_{i}X_{i}+\sum_{i}\beta_{ii}X_{i}^{2}+\sum_{i}{<}_{j}\beta_{ij}X_{i}X_{j}+\epsilon$$

was fitted to the experimental data of each strain, where Y is the predicted zone of inhibition, X_i_ are the coded independent variables, β_0_ is the intercept, β_i_ are linear coefficients, β_ii_ are quadratic coefficients, β_ij_ are two-factor interaction coefficients, and ϵ is the residual error.

### 2.5 Statistical analysis and modeling

Regression coefficients, analysis of variance (ANOVA) and response-surface and contour plots were obtained using Minitab statistical software, version 14.13. Goodness of fit was assessed by the coefficient of determination (R²), the adjusted R² and the predicted R², and by the lack-of-fit test against pure error from replicated center points. Effects were considered statistically significant at p < 0.05. The optimum combination of supplements for each strain was identified by desirability-based optimization of the fitted polynomial. Given that the models showed a significant lack-of-fit and a predicted R² of 0.00% (Section 3.3), the desirability optimization was used only to locate an approximate optimum region for each strain, and not as a validated quantitative predictor of the response at any single ingredient combination; the model-based predicted responses reported below are therefore presented as indicative point estimates accompanied by the corresponding limitations. Where appropriate, fitted-model diagnostics were also expressed in the present manuscript by recomputing the response from the coded coefficients and confirming agreement with the originally published R² values.

### 2.6 Validation under combined optimal conditions

To test whether the supplementation optima identified here combine constructively with previously published optimal culture conditions, validation fermentations were carried out at the desirability-defined supplement levels for each strain together with the prior optimal culture conditions (1.9% NaCl, 31 °C, initial pH 6.0). Bacteriocin activity was measured as described in Section 2.3 and compared with both the supplementation-only model prediction and the validation outcome obtained in the earlier culture-conditions study. Because validation was performed at the single predicted-optimum concentration for each strain, rather than across a gradient of concentrations around the optimum, it can confirm the presence of an improved response but cannot, on its own, delineate the width or robustness of the optimum region; this limitation is discussed in Section 4.5.

### 2.7 Non-parametric re-analysis and predictive validation

To assess predictive capacity independently of the second-order polynomial, and in response to the reviewers’ request, the 20-run dataset of each strain was additionally modeled with non-parametric and machine-learning regressors: Gaussian-process regression (radial-basis kernel with a white-noise term), support-vector regression (radial-basis kernel), k-nearest-neighbor regression, and random-forest and gradient-boosted-tree ensembles. Predictive performance was quantified by leave-one-out cross-validation (LOOCV), reporting the cross-validated coefficient of determination (Q²), the root-mean-square error (RMSE) and the mean absolute error (MAE), with the ordinary-least-squares second-order polynomial as a baseline. Predictors were standardized within each cross-validation fold to prevent information leakage, and the optimum of each fitted surface was located by exhaustive search over a dense grid spanning the design region. Analyses were performed in Python 3.12 with scikit-learn 1.8; the code is provided as **Supplementary Material**.

## 3 Results

### 3.1 Experimental design, observed responses and model fit

The CCD matrix and the experimentally observed zones of inhibition for both isolates are presented in **Table 1**. For both organisms, the largest experimental responses were obtained at and around the center point of the design (zones of 12.5-13.2 mm for *L. lactis* and 12.2-12.6 mm for *L. fermentum*), whereas the most extreme factorial points and the upper axial of glucose gave the lowest activities. The single highest experimental zone (13.62 mm for *L. lactis* at the low axial of glucose, Std 9) corresponds to a reduced-glucose point, foreshadowing an optimum-seeking quadratic dependence on glucose described below.

**Table 1:** Central composite design matrix (uncoded factor levels, by standard order) and experimental zone of inhibition for *L. lactis* MT186647 and *L. fermentum* MT186598 against S. aureus ATCC 19095.

Std	Glucose (g/L)	Sodium citrate (g/L)	KCl (g/L)	ZI L. lactis (mm)	ZI L. fermentum (mm)
1	5	0.3	0.55	10.22	9.85
2	10	0.3	0.55	9.35	8.92
3	5	1.0	0.55	9.23	9.66
4	10	1.0	0.55	9.44	8.12
5	5	0.3	3.0	10.85	10.20
6	10	0.3	3.0	9.88	9.02
7	5	1.0	3.0	9.87	9.12
8	10	1.0	3.0	9.28	8.87
9	3.3	0.65	1.78	13.62	12.89
10	11.7	0.65	1.78	9.12	8.65
11	7.5	0.06	1.78	12.21	12.33
12	7.5	1.24	1.78	10.14	10.22
13	7.5	0.65	1.78	12.55	12.11
14	7.5	0.65	3.84	10.98	10.21
15	7.5	0.65	1.78	12.85	12.22
16	7.5	0.65	1.78	12.83	12.58
17	7.5	0.65	1.78	13.21	12.56
18	7.5	0.65	1.78	12.71	12.52
19	7.5	0.65	1.78	12.98	12.35
20	7.5	0.65	1.78	12.54	12.40

Values are means of duplicate fermentations; the coefficient of variation between duplicates did not exceed 4.5% in any run.

**Figure 1:**
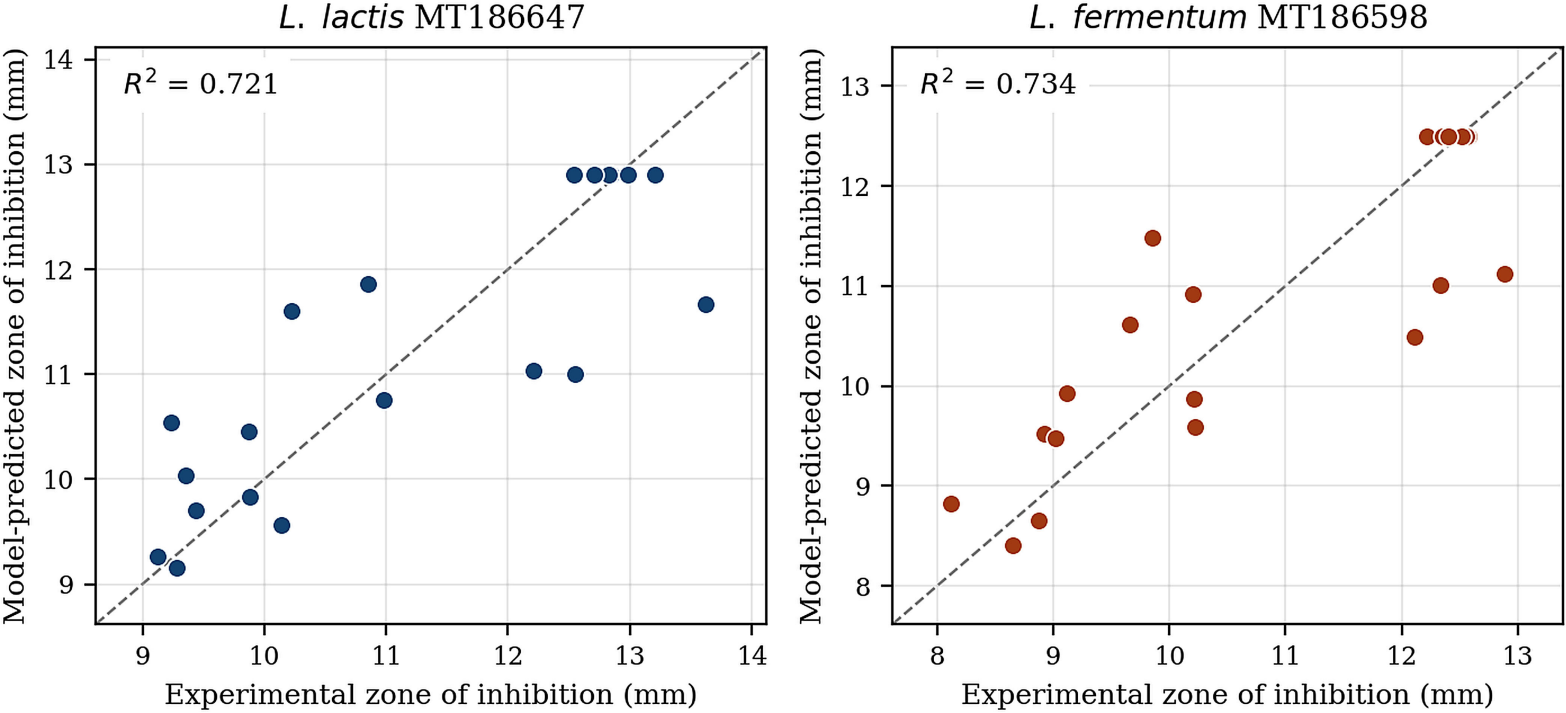
Parity (actual vs predicted) plots of bacteriocin activity for L. lactis MT186647 (left, blue) and L. fermentum MT186598 (right, orange). Predicted values were recomputed from the published coded coefficients; the dashed line is the line of equality, and R² values are computed from the points shown.

The agreement between the experimental zones and the model-predicted zones, computed by recomputing the response from the coded coefficients, is shown in **Figure 1**. For both organisms, the points are distributed evenly around the line of equality, and the recomputed R² values (0.721 for *L. lactis*; 0.734 for *L. fermentum*) match the originally reported R² values (71.85% and 73.15%) to within rounding, confirming that the published coefficients correctly describe the fitted models. The largest residuals are concentrated in the higher-response region, which is consistent with the significant lack-of-fit reported in Section 3.3.

### 3.2 Regression models and coefficient analysis

**Figure 2:**
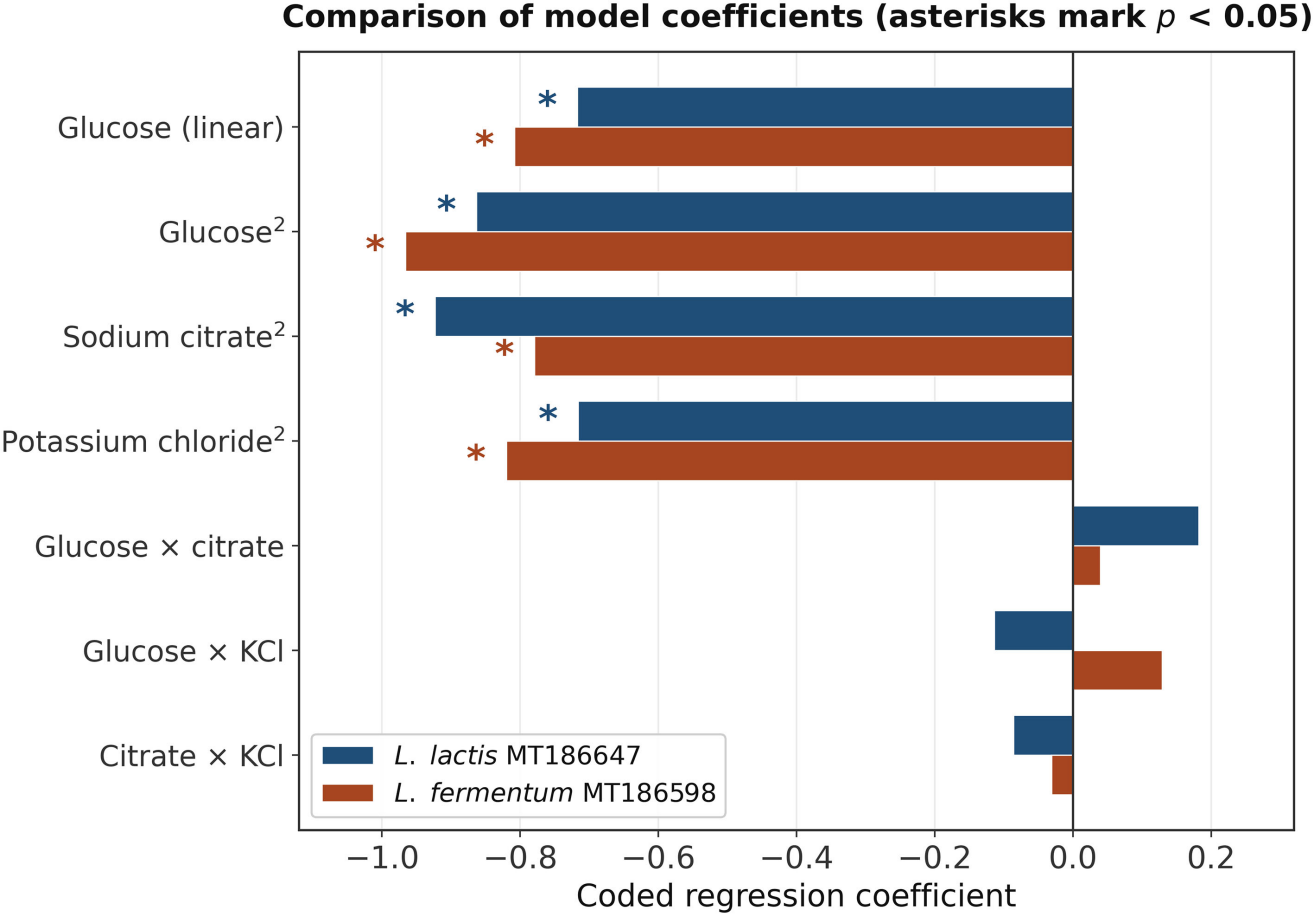
Side-by-side comparison of coded regression coefficients for the supplementation of bacteriocin production by L. lactis MT186647 and L. fermentum MT186598. Asterisks mark terms significant at p < 0.05. Negative quadratic coefficients of comparable magnitude across both strains indicate convex, optimum-seeking surfaces in all three factors.

The coded regression coefficients for both strains are presented in **Tables 2** and compared graphically in **Figure 2**, while the corresponding ANOVA results are shown in **Tables 3**, **4**. For both isolates, the linear term of glucose and the three quadratic terms (glucose², citrate² and KCl²) reached statistical significance (p < 0.05), while the linear terms of sodium citrate and potassium chloride and all three two-factor interactions did not (**Table 2**). The sign of all significant coefficients was negative, indicating that the response surface curves downwards from its interior maximum and that each significant factor exhibits an optimum-seeking quadratic dependence within the design region. .

**Table 2:** Coded regression coefficients and their p-values for the supplementation models of both isolates. Asterisks denote terms significant at p < 0.05.

Term	*L. lactis* Coeff.	*L. lactis* p	*L. fermentum* Coeff.	*L. fermentum* p
Glucose (linear)	−0.717	0.041 *	−0.808	0.030 *
Glucose²	−0.863	0.020 *	−0.966	0.012 *
Sodium citrate²	−0.923	0.014 *	−0.779	0.032 *
Potassium chloride²	−0.716	0.044 *	−0.820	0.025 *
Glucose × citrate	0.182	0.671	0.040	0.926
Glucose × KCl	−0.114	0.790	0.129	0.766
Citrate × KCl	−0.086	0.842	−0.031	0.944

Asterisks indicate statistically significant coefficients at p < 0.05.

**Table 3:** ANOVA for the L. lactis MT186647 supplementation model.

Source	DF	Adj SS	Adj MS	F	p
Model	9	35.5971	3.9552	2.84	0.060
Linear	3	9.6806	3.2269	2.31	0.138
Square	3	25.4855	8.4952	6.09	0.013
2-way interaction	3	0.4295	0.1432	0.10	0.957
Error	10	13.9445	1.3945		
Lack-of-fit	5	13.6820	2.7364	52.12	<0.001
Pure error	5	0.2625	0.0525		
Total	19	49.5416			

Model summary: S = 1.18087; R² = 71.85%; adjusted R² = 46.52%; predicted R² = 0.00%.

**Table 4:** ANOVA for the L. fermentum MT186598 supplementation model.

Source	DF	Adj SS	Adj MS	F	p
Model	9	38.6672	4.2964	3.03	0.050
Linear	3	11.8153	3.9384	2.78	0.096
Square	3	26.7147	8.9049	6.28	0.011
2-way interaction	3	0.1528	0.0509	0.04	0.990
Error	10	14.1908	1.4191		
Lack-of-fit	5	14.0923	2.8185	143.09	<0.001
Pure error	5	0.0985	0.0197		
Total	19	52.8580			

Model summary: S = 1.19125; R² = 73.15%; adjusted R² = 48.99%; predicted R² = 0.00%.

The two strains differed quantitatively in the relative magnitude of their coefficients. *L. fermentum* showed a slightly larger linear coefficient for glucose (−0.808 vs −0.717) and stronger quadratic curvatures for glucose and KCl (Glucose² −0.966 vs −0.863; KCl² −0.820 vs −0.716), whereas *L. lactis* showed a stronger quadratic curvature in sodium citrate (Citrate² −0.923 vs −0.779). The interaction terms were small and non-significant for both organisms (coefficient ≤ 0.20 in absolute value; **Table 2**), so the models resolve only the linear and quadratic structure of the response and do not provide evidence for factor interactions.

### 3.3 Analysis of variance and model adequacy

The overall regression model was only marginally significant for *L. fermentum* (p = 0.050) and non-significant at the conventional threshold for *L. lactis* (p = 0.060) at the conventional α = 0.05 (**Tables 3**, **4**). In both ANOVAs, the squared block of terms was the dominant source of explained variation, contributing F-ratios of 6.09 (*L. lactis*) and 6.28 (*L. fermentum*) at p = 0.013 and p = 0.011, respectively. The linear block was non-significant overall for both isolates, dominated by the single significant glucose term. The two-factor interaction block was non-significant in both fits, with F-ratios near zero. The *L. lactis* and *L. fermentum* models accounted for 71.85% and 73.15% of the variation in zone of inhibition, with adjusted R² values of 46.52% and 48.99% and predicted R² values of 0.00% in both cases; the lack-of-fit term was highly significant in both fits (p < 0.001). This combination of findings requires cautious interpretation. The significant quadratic block establishes that the response is curved in each factor and that an interior optimum exists, but the marginal overall model significance, the modest adjusted R² (46-49%), the predicted R² of 0.00% and the highly significant lack-of-fit together indicate that the fitted second-order polynomials do not reliably predict the response at new, un-run factor combinations. Accordingly, the models are used in what follows only to locate an approximate optimum region, and the model-derived predicted responses (Sections 3.4-3.5) are reported as indicative estimates rather than validated predictions. The statistical and design remedies that would be required to obtain a genuinely predictive model are discussed in Section 4.5.

### 3.4 Response surface and contour analysis

**Figure 3:**
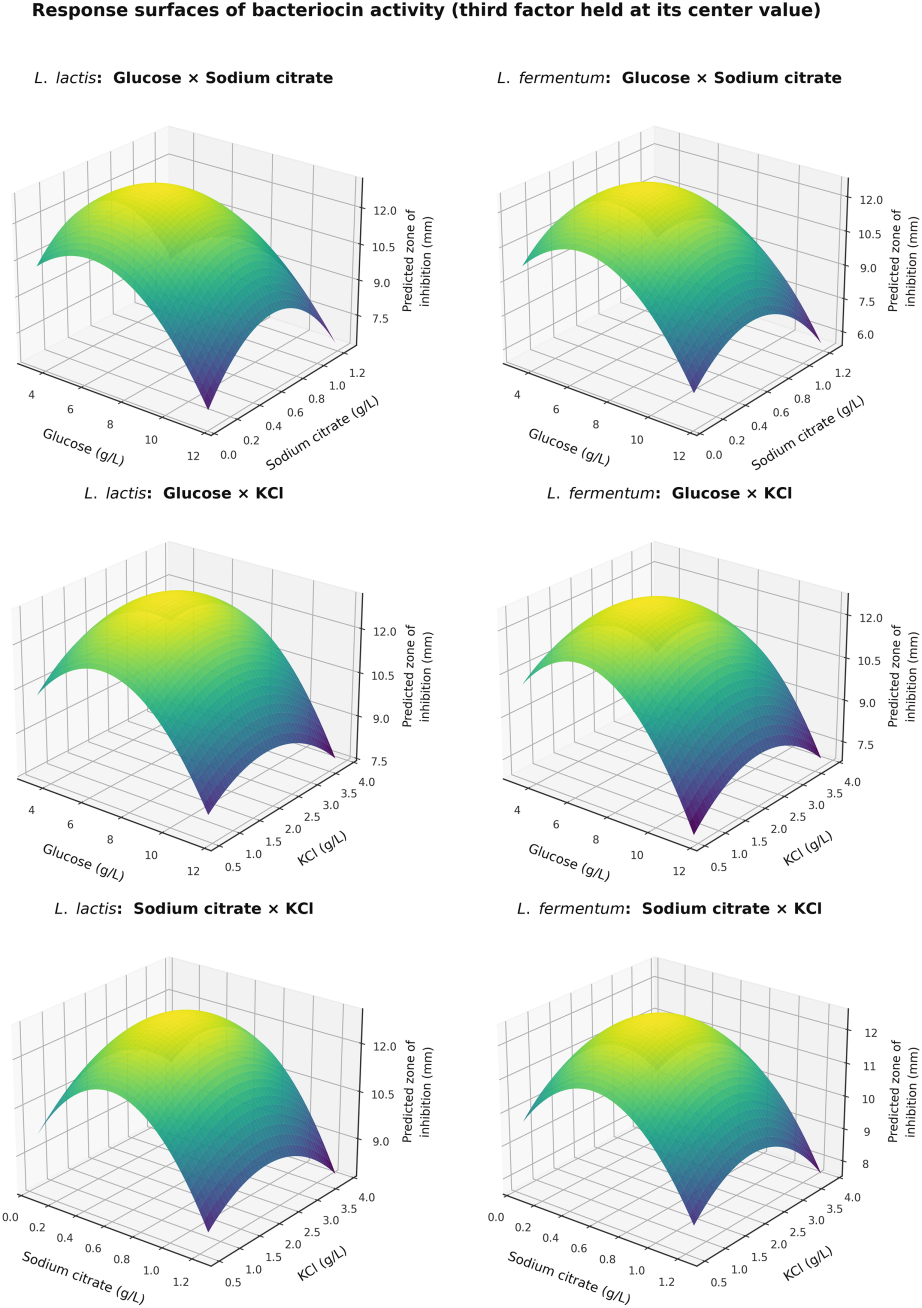
Three-dimensional response surfaces of bacteriocin activity as a function of the three supplements, for L. lactis MT186647 (left column) and L. fermentum MT186598 (right column). In each row, the third factor was held at its center value. The surfaces curve downwards from interior maxima for both strains, reflecting the significantly negative quadratic coefficients of all three supplements.

**Figure 4:**
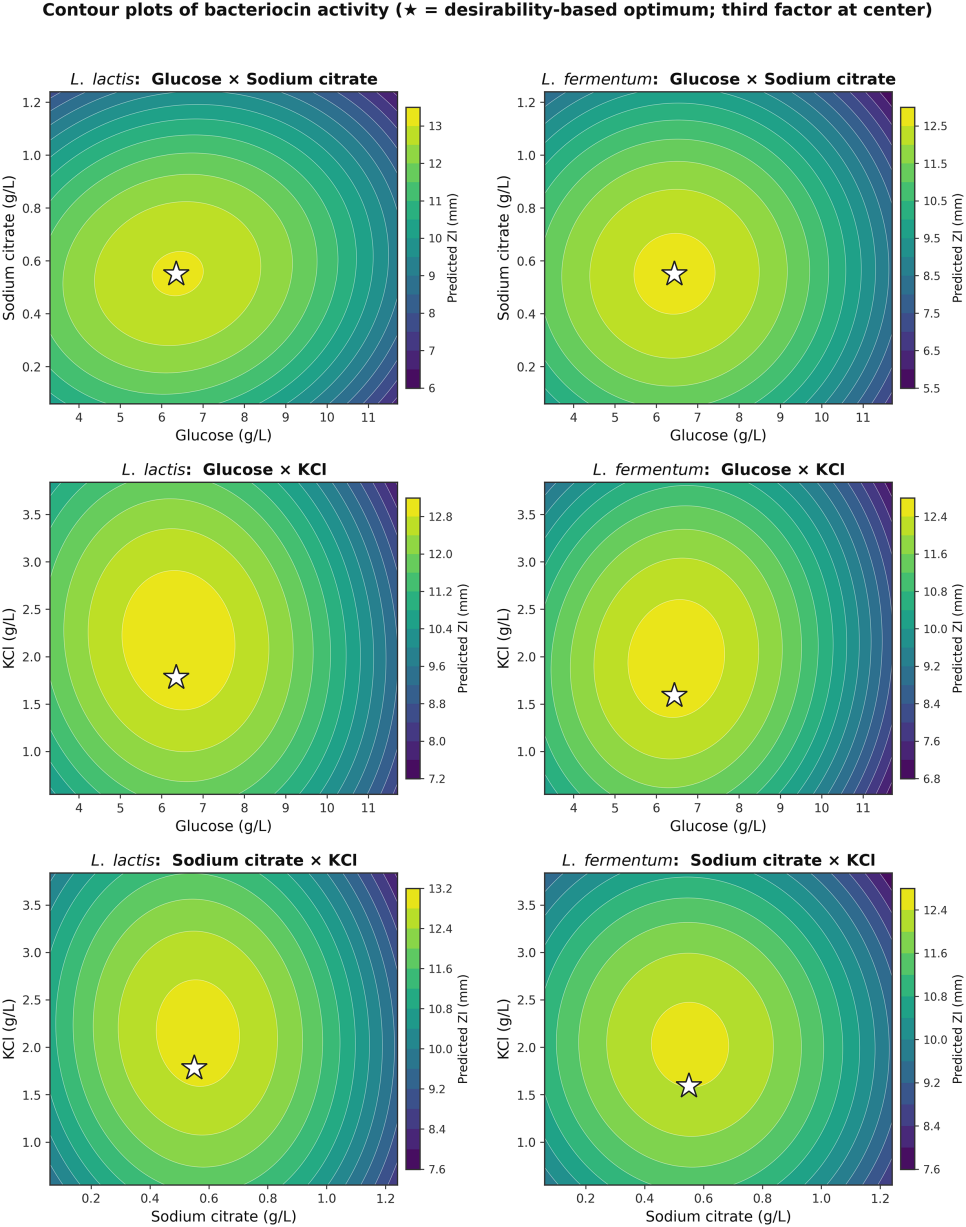
Two-dimensional contour plots corresponding to the surfaces of **Figure 3**. The white star marks the desirability-based optimum projected onto each plane (L. lactis: 6.35 g/L glucose, 0.55 g/L sodium citrate, 1.78 g/L potassium chloride; L. fermentum: 6.44 g/L glucose, 0.55 g/L sodium citrate, 1.59 g/L potassium chloride). Concentric, near-circular contours reflect the near-absence of factor interactions.

The three-dimensional response surfaces (**Figure 3**) and two-dimensional contour plots (**Figure 4**) illustrate the joint effects of the three supplements on bacteriocin production. For both organisms, the surface over the glucose-citrate plane (with KCl fixed at the center) is a dome with an apex displaced slightly toward lower-than-center glucose and lower-than-center citrate; the glucose-KCl plane is similarly dome-shaped, with the apex close to the center value of KCl for *L. lactis* and at slightly lower KCl for *L. fermentum*; the citrate-KCl plane shows a comparatively flatter optimum centered near the design centroid. The shapes of the contours are concentric and elliptical without strong tilt, consistent with the small and non-significant interaction coefficients (**Table 2**). The strain-specific optima identified by desirability analysis lie within the elliptical maxima in every plane, confirming that the optima are interior points of the design region and not at its boundary. Because the underlying models have zero predicted R² and a significant lack-of-fit, these surfaces should be read as a qualitative map of the optimum region rather than as an exact quantitative surface.

### 3.5 Optimum prediction and combined-medium validation

Desirability-based optimization of the two models yielded similar but non-identical optima (**Table 5**). The optimum glucose and sodium citrate levels were almost interchangeable between the two organisms (6.35 vs 6.44 g/L glucose; 0.55 vs 0.55 g/L sodium citrate), whereas the optimum potassium chloride level was slightly higher for *L. lactis* (1.78 g/L) than for *L. fermentum* (1.59 g/L). The model-estimated zone of inhibition at the optimum region was 13.12 mm for *L. lactis* (overall desirability = 0.89) and 12.74 mm for *L. fermentum* (overall desirability = 0.97). In view of the zero predicted R² and significant lack-of-fit, these values should be read as central estimates within the optimum region rather than as validated point predictions.

**Table 5:** Predicted optimum supplement concentrations, supplementation-only estimated activity, and validation activity obtained when the supplement optima were combined with the previously established optimal culture conditions (1.9% NaCl, 31 °C, initial pH 6.0).

Parameter	*L. lactis* MT186647	*L. fermentum* MT186598
Optimum glucose (g/L)	6.35	6.44
Optimum sodium citrate (g/L)	0.55	0.55
Optimum potassium chloride (g/L)	1.78	1.59
Desirability	0.89	0.97
Estimated ZI, supplements only (mm)	13.12	12.74
Prior culture-conditions-only outcome (mm)	13.33	11.75
Validation ZI, combined optimal medium (mm)	15.22 ± 0.68	14.96 ± 0.67
Gain vs supplementation-only estimate	+16.0%	+17.4%
Gain vs prior culture-conditions outcome	+14.2%	+27.3%

Validation values are means ± SD of duplicate fermentations (the coefficient of variation did not exceed 4.5%).

**Figure 5:**
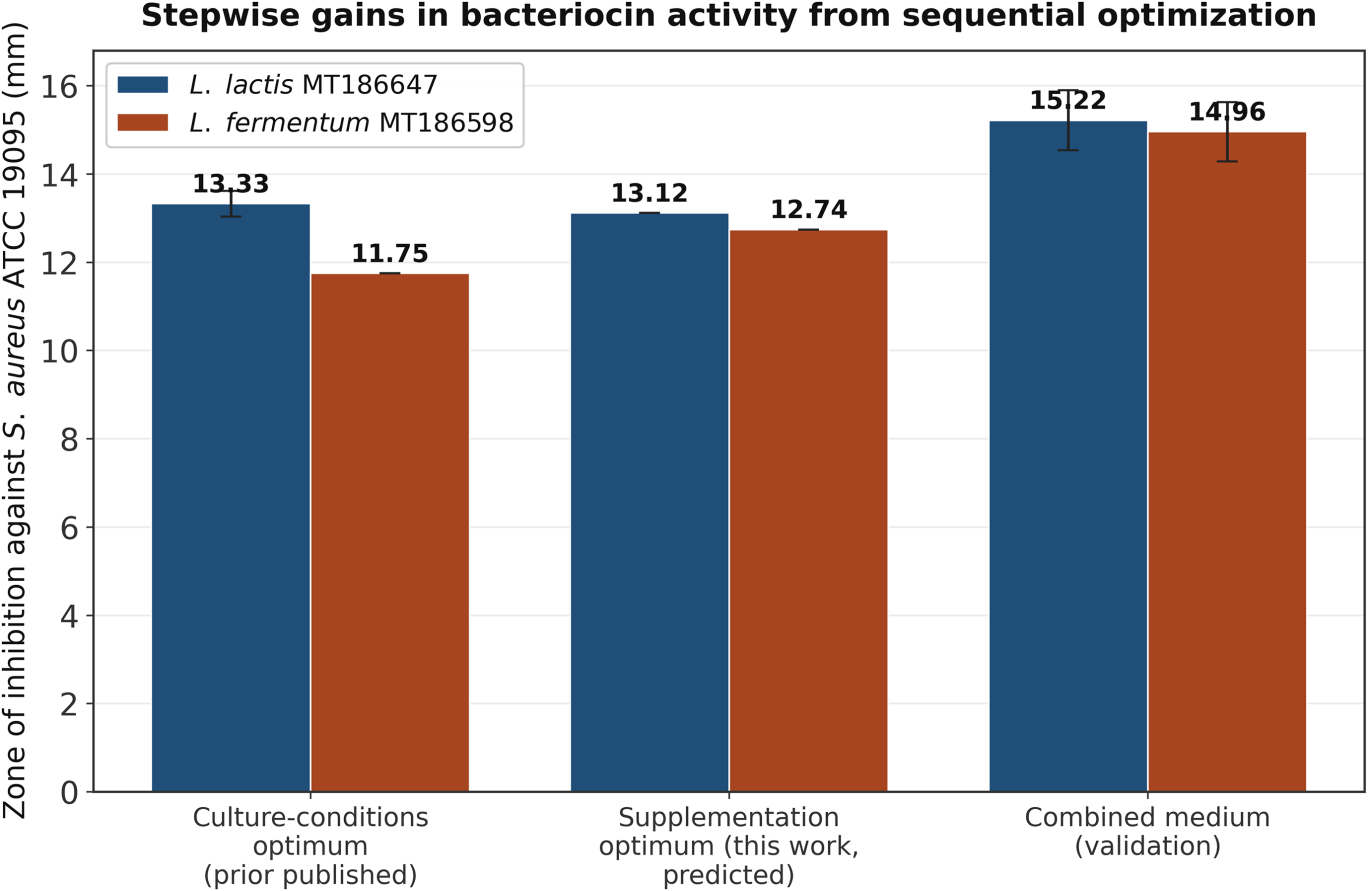
Stepwise gains in bacteriocin activity from sequential optimization. The combined-medium validation step (right) exceeds both the prior culture-conditions outcome (left) and the supplementation-only estimate (middle), and the relative gain is larger for L. fermentum than for L. lactis. Error bars on this study’s combined-medium validation reflect a coefficient of variation of 4.5% between duplicate fermentations; the error bar on the prior L. lactis culture-conditions point is the published SD (± 0.29 mm); other bars are model-estimated point values.

Validation fermentations carried out at the supplement optima combined with the previously established optimal culture conditions (1.9% NaCl, 31 °C, initial pH 6.0) produced zones of inhibition of 15.22 ± 0.68 mm (*L. lactis*) and 14.96 ± 0.67 mm (*L. fermentum*) (means of duplicate fermentations; the coefficient of variation between duplicates did not exceed 4.5% in any run). These validation values exceed both the supplementation-only model estimates (by 16.0% and 17.4%, respectively) and the culture-condition-only outcomes reported in our earlier publications (by 14.2% and 27.3%; **Figure 5**), indicating a directional improvement when the two optimization layers are applied together. Two cautions attach to this comparison. First, the percentage gains were not subjected to a formal significance test (e.g., a t-test against the validation replicates), so they describe an observed directional improvement rather than a statistically confirmed synergy. Second, the validation medium additionally contained 1.9% NaCl, a strong ionic factor that was optimized in the earlier culture-conditions study but was fixed and therefore not represented in the present supplement CCD; part of the excess of the validation activity over the supplement-only estimate is therefore expected to reflect the main effect of this added NaCl rather than an interaction captured by the supplement-only model. A single-batch, single-concentration validation cannot separate these contributions, and confirmatory trials across a concentration gradient are recommended (Section 4.5).

### 3.6 Predictive re-analysis by machine-learning regression

To test predictive capacity independently of the second-order polynomial, and following the reviewers’ request, the 20-run dataset of each strain was re-analyzed with five non-parametric and machine-learning regressors under leave-one-out cross-validation (LOOCV); full details are given in Section 2.7 and the **Supplementary Material**. The cross-validated coefficient of determination (Q²) measures the ability to predict a run withheld from fitting, and is therefore a direct test of the predictive concern raised by the significant lack-of-fit.

**Figure 6:**
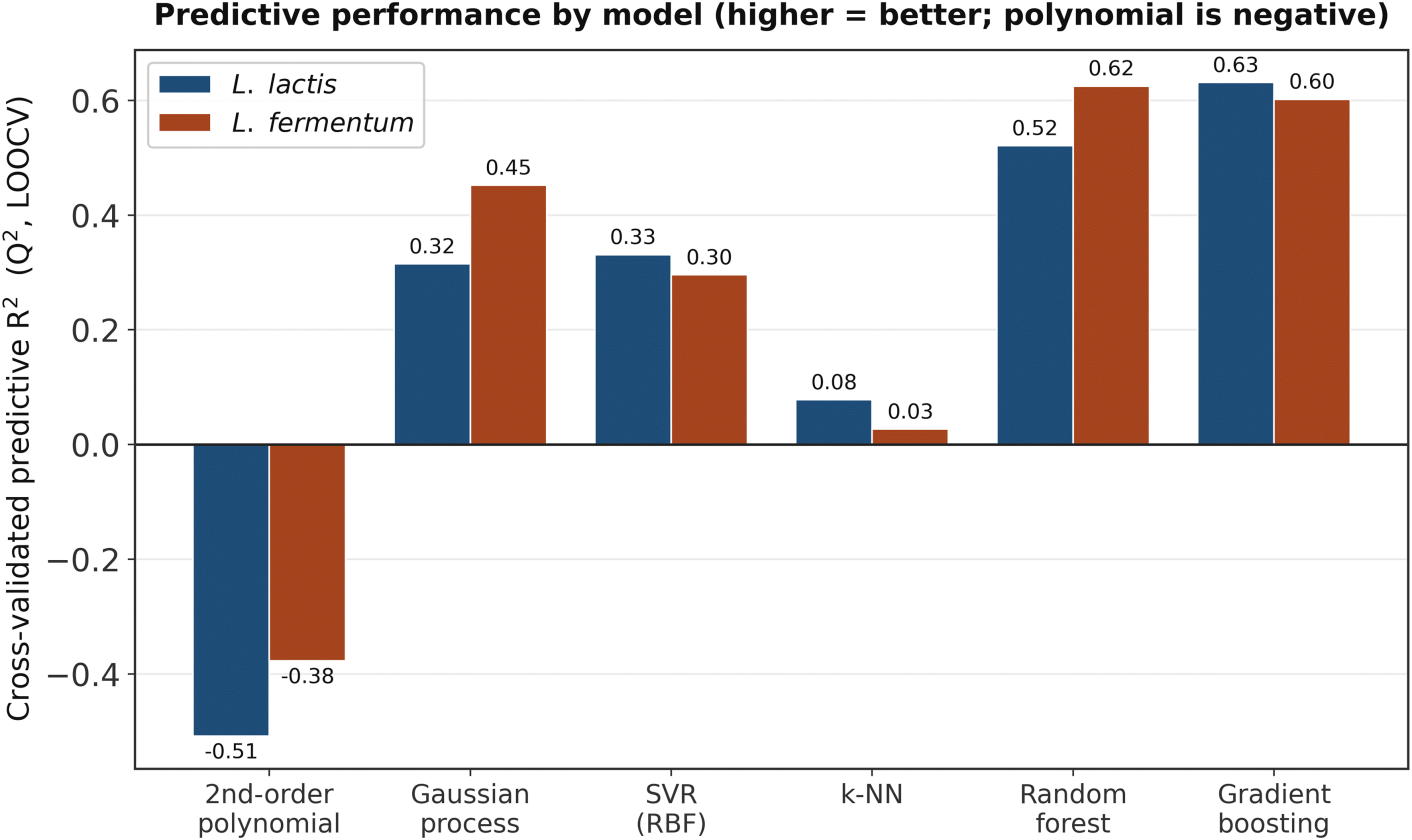
Cross-validated predictive R² (Q², LOOCV) by model. The second-order polynomial is negative for both strains, whereas the tree ensembles and Gaussian process regression are positive.

**Figure 7:**
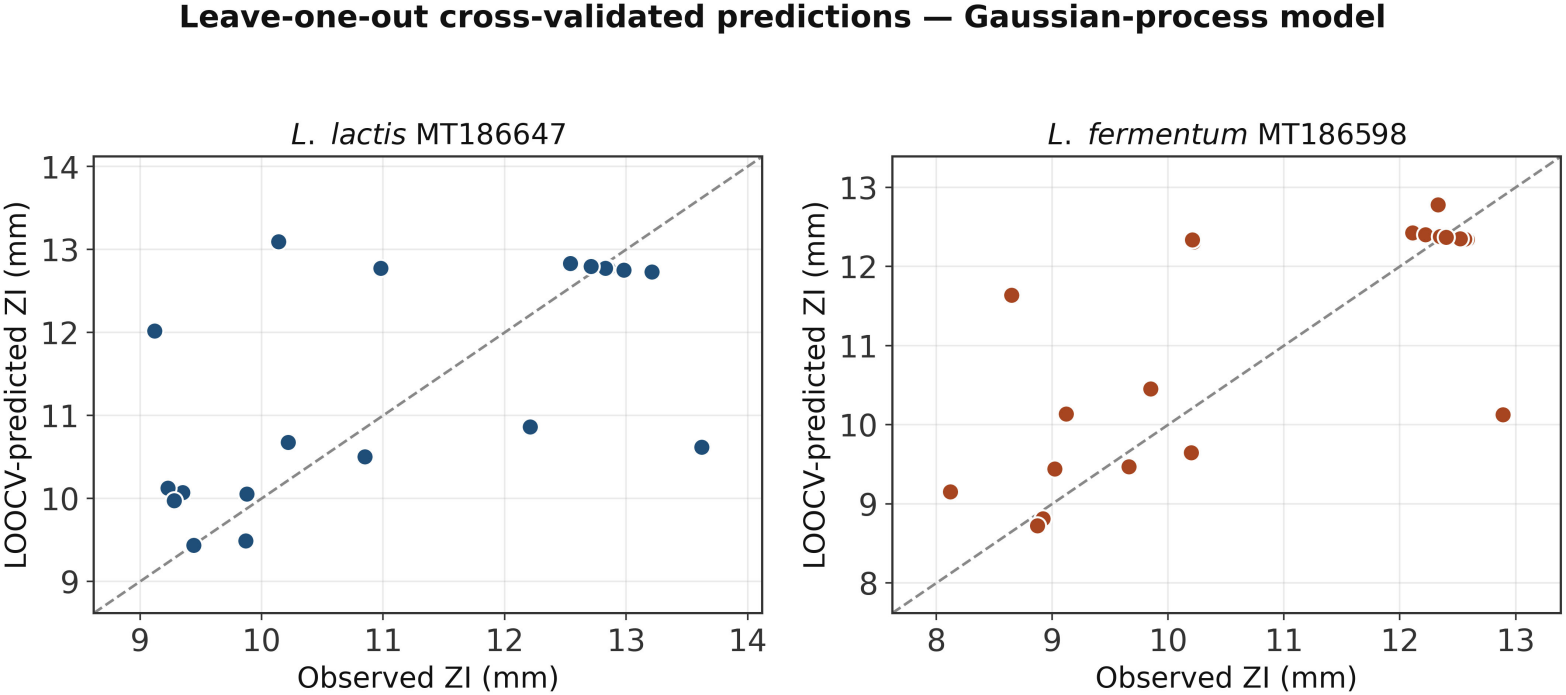
Leave-one-out cross-validated predictions versus observed zones of inhibition for the Gaussian-process model (L. lactis, left; L. fermentum, right). Points scatter around the line of equality without systematic bias.

**Table 6** reports the results. Consistent with the significant lack-of-fit in Section 3.3, the second-order polynomial gave a negative cross-validated Q² for both strains (−0.51 for *L. lactis*; −0.38 for *L. fermentum*), meaning that it predicts a withheld run less accurately than the overall mean and therefore has effectively no out-of-sample predictive power, despite its high in-sample R². The non-parametric models recovered a modest but real predictive signal: the best cross-validated Q² was 0.63 (gradient boosting) for *L. lactis* and 0.63 (random forest) for *L. fermentum*, with Gaussian-process regression giving 0.32 and 0.45, respectively (**Figures 6**, **7**).

**Table 6:** Leave-one-out cross-validated predictive performance of the fitted models.

Model	R² (in) L. lactis	Q² (LOOCV) L. lactis	R² (in) L. fermentum	Q² (LOOCV) L. fermentum
2nd-order polynomial (baseline)	0.842	−0.508	0.859	−0.377
Gaussian process (RBF + noise)	0.993	0.315	0.996	0.452
Support vector regression (RBF)	0.990	0.331	0.994	0.296
k-nearest neighbors (k = 3)	0.992	0.078	0.995	0.027
Random forest	0.938	0.521	0.951	0.625
Gradient boosting	0.989	0.631	0.987	0.602

R² (in-sample) is the ordinary fit; Q² (LOOCV) is the cross-validated predictive R². A negative Q² denotes no out-of-sample predictive power.

The optimum located by these more flexible models was, however, not stable across model families (**Table 7**): the predicted-optimum glucose level ranged from 3.3 to 7.4 g/L and the potassium chloride level from 0.55 to 1.83 g/L, with several optima falling on the boundary of the sampled region - notably the lower potassium chloride bound, for which the design contained no low axial point. This instability shows that the present 20-run design, although sufficient to establish curvature and an approximate optimum region, is too sparse for a flexible model to locate a robust optimum on its own.

**Table 7:** Optimum located by grid search over the design region for each model.

Model	*L. lactis* optimum (glucose/citrate/KCl g/L → mm)	*L. fermentum* optimum (glucose/citrate/KCl g/L → mm)
2nd-order polynomial (original)	6.35/0.55/1.78 → 13.12	6.44/0.55/1.59 → 12.74
Gaussian process	5.19/0.64/0.55 → 14.21	7.41/0.42/0.55 → 13.17
Support vector regression	4.16/0.59/1.83 → 13.61	5.70/0.57/1.83 → 13.06
Random forest	3.30/0.49/1.83 → 13.05	6.39/0.49/1.22 → 12.39
Gradient boosting	3.30/0.49/1.22 → 13.60	3.30/0.49/1.22 → 12.87

Values on a sampled boundary (glucose lower axial 3.30 g/L;potassium chloride lower bound 0.55 g/L, which has no low axial point)indicate extrapolation rather than a resolved optimum.

These cross-validated results indicate that the second-order polynomial has no out-of-sample predictive power (negative Q²), whereas the non-parametric models recover a modest predictive signal (Q² ≈ 0.5–0.6); the located optimum, however, varies across model families and in several cases falls on the boundary of the sampled region.

## 4 Discussion

### 4.1 Carbon limitation rather than carbon excess favors bacteriocin output

The most consistent finding of this study is that, for both organisms, bacteriocin yield rises to a maximum at a glucose concentration of approximately 6.4 g/L and falls at both lower and higher levels, with the linear coefficient of glucose being significant and negative and its quadratic coefficient being significantly negative for both strains. This pattern is consistent with a carbon-limited rather than carbon-rich optimum, and may be interpreted on at least three grounds. First, an excess of readily fermentable carbohydrate provokes carbon catabolite repression in LAB, redirecting flux toward growth and homofermentative or mixed-acid acidification at the expense of secondary peptide synthesis (Vázquez et al., 2004; Abbasiliasi et al., 2017; Dorau et al., 2021). Second, intense acidification at high glucose can carry the pH below the range optimal for bacteriocin secretion or stability (Bogovic Matijašić et al., 2001; Juarez Tomás et al., 2002); the model in the present work does not include pH as a factor, but the producer cultures were initiated at pH 6.0 and not pH-controlled, so high glucose would be expected to drive the broth furthest from the favorable range during incubation. Third, at very low glucose, carbon and energy become limiting for biomass and therefore for bacteriocin, generating the rising left-hand limb of the quadratic. The intermediate optimum at moderate glucose reflects the balance between these effects. An important caveat applies to the second of these mechanisms: because the final pH of each run was not measured, the pH-mediated explanation remains a plausible hypothesis rather than a demonstrated cause. With pH fixed only at inoculation and left uncontrolled and unmonitored thereafter, the reduced activity at high glucose cannot be unambiguously partitioned into a direct carbon-catabolite-repression component and an indirect pH-driven component. Future runs should record the final pH (or include pH as an explicit design factor) so that these two contributions can be separated.

These findings are in broad agreement with previous RSM studies of bacteriocin-producing LAB. Kumar et al. (2012) reported a glucose optimum well below the highest level tested for *L. casei*, and Suganthi and Mohanasrinivasan (2015) likewise identified moderate carbohydrate levels as optimal for *P. pentosaceus* KC692718. Lee et al. (2012), working with *L. brevis* DF01, also identified a moderate carbon-source optimum, while Cladera-Olivera et al. (2004) showed for *B. licheniformis* P40 that even relatively complex carbohydrate sources (cheese whey) require an optimum-seeking adjustment rather than maximization. The convergence of these reports supports the general view that bacteriocin synthesis is favored under carbon-controlled rather than carbon-saturated conditions.

### 4.2 Sodium citrate: buffering and metabolic effects at an intermediate optimum

Sodium citrate was not significant as a linear factor in either model but exerted a strongly significant negative quadratic effect in both, indicating an intermediate optimum near 0.55 g/L. Several mechanisms can underlie this behavior. As a weak organic-acid buffer, citrate moderates the rapid acidification produced by LAB growth and extends the period over which the broth remains in the pH range conducive to bacteriocin synthesis (Onwuakor et al., 2014); too little citrate provides insufficient buffering, while too much can shift the pH above the LAB optimum and impose ionic stress. In addition, many LAB, particularly mesophilic lactococci, can metabolize citrate via the cit operon, with diacetyl and acetoin among the end-products (Castro et al., 2011); the net effect is strain-dependent and concentration-dependent, again favoring a moderate level. Finally, citrate chelates divalent cations such as Mg²^+^ and Mn²^+^, which are cofactors for enzymes involved in central metabolism; at high citrate concentrations, this chelation could limit enzyme activity. The fact that the optimum citrate concentration was identical for the two organisms despite their different physiologies suggests that the dominant mechanism in this system is a relatively non-specific one, most plausibly the buffering effect.

### 4.3 Potassium chloride: osmotic balance and a slight strain-specific optimum

Potassium is the major intracellular cation in bacterial cells and is required for osmotic balance, membrane potential and the activity of several enzymes (Himelbloom et al., 2001). At too low a concentration, intracellular potassium homeostasis cannot be maintained; at too high a concentration, ionic strength rises, and osmotic stress depresses growth and metabolism. The negative quadratic coefficient of KCl in both models is consistent with this dual-sided dependence. The optimum KCl level differed slightly between the two organisms, being higher for *L. lactis* (1.78 g/L) than for *L. fermentum* (1.59 g/L). Lactococci are generally more tolerant of higher salt levels than heterofermentative lactobacilli (Carr et al., 2002), and the small difference in the present optima is in keeping with that broader observation. It is also worth noting that the supplementation was carried out in addition to the optimal NaCl level identified in the culture-conditions work (1.9% w/v); the total ionic load on the producer cells in the validation experiments was therefore appreciable, and the modest KCl optimum is consistent with the additional Na^+^ load already present.

### 4.4 Strain-specific differences and the comparative value of a two-strain optimization

Although the qualitative response of the two organisms to supplementation is similar - negative linear effect of glucose, no significant linear effect of citrate or KCl, significant negative quadratic effects throughout, and negligible interactions - the quantitative differences are not negligible. *L. fermentum* showed stronger quadratic curvature in glucose and KCl, implying that its response is more sharply localised in those two factors and that deviations from its optimum will have a larger penalty; *L. lactis* showed stronger quadratic curvature in sodium citrate, implying a sharper citrate optimum for that organism. These differences are biologically interpretable: heterofermentative lactobacilli such as *L. fermentum* can metabolize glucose by both the Embden-Meyerhof and the phosphoketolase pathways, and the partitioning of carbon between them is sensitive to glucose level (Chapot-Chartier and Kulakauskas, 2014); citrate-metabolising lactococci such as *L. lactis* subspecies are sensitive to citrate concentration in a way that homofermentative-only organisms are not (Castro et al., 2011). The practical consequence is that, despite their similar optima, the two strains should be optimized using strain-specific models; a single average optimum would underperform in both.

Beyond the level of individual coefficients, the comparative design also strengthens the conclusions that can be drawn from each model. Two independent CCDs on two organisms have produced essentially the same qualitative landscape - dome-shaped surfaces with interior optima, negligible interactions, and dominant quadratic terms - which is a stronger argument for the underlying biology of bacteriocin-medium dependence than either dataset alone.

### 4.5 Model adequacy: significant curvature but limited predictive validity

Both models combine significant quadratic terms with zero predicted R² (0.00%), modest adjusted R² (46-49%), a highly significant lack-of-fit (p < 0.001) and only marginal overall model significance. We treat this as a genuine limitation of the fitted models rather than as an incidental feature of biological RSM. Its interpretation and its consequences for the study’s conclusions are set out below.

What the models do and do not support. The significance of the squared terms shows that the response is non-linear in each factor and that an interior optimum exists; the parity and contour plots confirm that the approximate location of the optimum region is identified consistently. However, a predicted R² of 0.00% together with a significant lack-of-fit means that the second-order polynomials have no demonstrated ability to predict the response of new, un-run factor combinations, and that the models therefore confirm the existence of curvature without precisely locating or quantifying the optimum. Desirability-based point predictions and any percentage-gain calculations derived from them must consequently be read as indicative of a region rather than as accurate point estimates, and the identified parameters represent a preferable concentration range (an interval estimate) rather than an exact global optimum (a point estimate).

Likely causes. This is most likely attributable to (i) experimental noise inherent to the agar well diffusion assay, in which zone diameter readings carry an irreducible measurement variance, compounded by the use of duplicate rather than triplicate replication, which limits the precision with which pure error, and hence lack-of-fit, can be estimated; (ii) unmodeled batch effects between runs, including the uncontrolled and unrecorded final pH discussed in Section 4.1; and (iii) the possibility that the true response surface contains higher-order curvature or local features that a second-order polynomial cannot capture (Sarabia and Ortiz, 2009), possibly combined with heteroscedasticity in the zone-diameter response.

Remedies for future work. Several standard remedies would be expected to improve model adequacy and were not applied here, and we recommend them for follow-up studies: (a) transforming the response (for example a logarithmic or Box-Cox transformation of the zone-diameter data) to stabilize the variance and reduce lack-of-fit; (b) augmenting the design with additional axial points and replicated center points, and moving from duplicate to triplicate fermentations, to sharpen the estimate of pure error; (c) considering higher-order (e.g., reduced cubic) terms where curvature is incompletely captured; and (d) validating across a gradient of concentrations around the predicted optimum (for example ±10% in each factor) rather than at a single point, so that the robustness and width of the optimum region can be assessed. Because the predicted R² is zero, the elevated reading at the single validation point cannot by itself exclude a contribution from random run-to-run variation, which reinforces the need for gradient validation. Where a smooth low-order polynomial proves inadequate, non-parametric or machine-learning regression frameworks (for example artificial neural networks or genetic-algorithm-assisted optimization) offer an alternative route to a predictive model on the same or an expanded dataset.

Bearing on the present conclusions. The practical implication is that the optima identified here should be regarded as best estimates of the region in which bacteriocin yield is maximized rather than as exact point-predictions of the zone diameter at any particular ingredient combination. The validation experiments are consistent with this interpretation: the observed activity at the predicted optima fell within the upper range of the response surface but exceeded the model estimate by 16-17%, a deviation that is itself a symptom of the models’ limited predictive accuracy and that, as noted in Section 3.5, also reflects the added NaCl not represented in the supplement design.

A non-parametric re-analysis (Section 3.6) reinforced this conclusion from both directions. Machine-learning regressors raised the cross-validated Q² from negative values for the polynomial to approximately 0.5-0.6, indicating that part of the variance treated as lack-of-fit is structured rather than pure noise and is recoverable with a more flexible model. At the same time, the optima identified by these models were unstable and tended to migrate to sparsely sampled design boundaries, which shows that improved modeling cannot substitute for improved sampling. Taken together, these results argue that the most reliable route to a predictive optimum is an augmented design, after which either a transformed-response polynomial or a Gaussian-process model could be fitted with genuine predictive validity.

Two implications follow. The re-analysis confirms that the second-order models should be used only to locate an approximate optimum region, not for quantitative prediction. Obtaining a predictively valid optimum will require an augmented design, additional runs around the current optimum region together with the missing low potassium-chloride axial point, in triplicate and with final pH recorded, after which either a transformed-response polynomial or a Gaussian-process model could be fitted with genuine predictive validity.

### 4.6 Combined supplementation and culture-conditions optima: an additive, not formally synergistic, improvement

Perhaps the most practically useful finding of this study is that the supplement optima identified here, when combined with the previously published optimal culture conditions (1.9% NaCl, 31 °C, initial pH 6.0) according to Onwuakor et al. (2020), produced bacteriocin activity that exceeded either set of optima taken on its own (**Figure 5**). For *L. lactis*, the validation activity (15.22 mm) exceeded the culture-conditions-only validation (13.33 mm) by 14.2% and exceeded the supplementation-only estimate (13.12 mm) by 16.0%. For *L. fermentum*, the validation activity (14.96 mm) exceeded the culture-conditions-only outcome (11.75 mm predicted in the earlier study) by 27.3% and the supplementation-only estimate (12.74 mm) by 17.4%. Both gains are positive, which shows that the two sets of optimized variables - physical environment on the one hand, medium composition on the other - do not act antagonistically. This should, however, be interpreted conservatively. The comparison rests on model-based estimates for one arm and single-batch validation for the other, and the percentage gains were not tested for statistical significance; the data therefore support an additive improvement but do not, on their own, establish a formally significant synergy.

A specific caveat concerns the role of NaCl. The supplement-only model estimates 13.12 mm for *L. lactis*, whereas the validation medium, which additionally contained 1.9% NaCl, yielded 15.22 mm. Because NaCl is a strong ionic factor that was optimized in the earlier culture-conditions study but was fixed and excluded from the present supplement CCD, a substantial part of the excess activity is most plausibly the main effect of that added NaCl rather than an interaction between NaCl and the three supplements. The present supplement-only design cannot capture this contribution, and this is a further reason why the combined-medium gain should be described as additive rather than synergistic. It also identifies the most valuable next experiment: an expanded design that includes NaCl (and ideally temperature and pH) alongside the three supplements, so that the ionic and nutritional factors can be modeled jointly and any genuine interactions detected. The proportional gain was larger for *L. fermentum*, suggesting that this organism may be more responsive to integrated optimization than its lactococcal counterpart, although this observation, too, awaits confirmation in replicated, statistically tested trials.

### 4.7 Limitations, implications for biopreservation and starter-culture development

Design and analysis limitations. Three limitations should be weighed alongside the applied implications below. First, the overall optimization strategy was sequential and compartmentalized: culture conditions were optimized in earlier work and the medium supplements here, with the two brought together only at the validation stage. A more rigorous strategy would integrate all critical factors (glucose, citrate, KCl, NaCl, temperature and pH) into a single expanded CCD or Box-Behnken design, or follow a formal sequential route (Plackett-Burman screening → steepest-ascent path → CCD). Second, the fitted models showed a significant lack-of-fit and zero predicted R² (Section 4.5), so the identified optima define a preferable region rather than an exact global optimum. Third, validation was performed at a single concentration per strain and in duplicate; multi-point, triplicate validation is needed to confirm robustness. We present the applied implications below within these constraints.

From an applied perspective, the supplements employed here are inexpensive and food-grade, and the absolute concentrations identified (6.4 g/L glucose, 0.55 g/L sodium citrate, 1.6-1.8 g/L potassium chloride) are well within levels normally tolerated in fermented-food matrices. The combined-optimum activity of approximately 15 mm against *S. aureus*, achieved by both isolates under the same culture conditions, supports their further evaluation as defined starter or protective cultures for the controlled fermentation of *P. macrophylla*. Because both organisms reach similar peak activities under similar optima, they can, in principle, be used together in a co-culture starter, an approach previously shown in our work to enhance B-vitamin biosynthesis on the same substrate. Beyond *P. macrophylla*, the additive composition is sufficiently generic to be tested in other protein-rich African fermented foods. Two important caveats apply: the activity is reported against a single Gram-positive indicator (*S. aureus* ATCC 19095), so spectrum-broadening trials against Gram-negative and additional Gram-positive pathogens are needed; and the limited predictive validity of the models indicates that confirmatory fermentations at and around the optima should be conducted before scale-up.

## 5 Conclusion

Response surface methodology based on a three-factor central composite design was used to optimize the supplementation of an African oil bean seed broth with glucose, sodium citrate and potassium chloride for enhanced bacteriocin production by *Lactococcus lactis* MT186647 and *Lactobacillus fermentum* MT186598. For both isolates, glucose exerted a significant negative linear effect, and all three supplements exerted significant negative quadratic effects, with negligible interactions, defining dome-shaped response surfaces with interior optima. The desirability-based optima were 6.35 g/L glucose, 0.55 g/L sodium citrate and 1.78 g/L potassium chloride for *L. lactis* (estimated activity 13.12 mm) and 6.44 g/L glucose, 0.55 g/L sodium citrate and 1.59 g/L potassium chloride for *L. fermentum* (estimated activity 12.74 mm). Validation fermentations carried out at these optima in combination with the previously established optimal culture conditions (1.9% NaCl, 31 °C, initial pH 6.0) produced bacteriocin activity of 15.22 mm (*L. lactis*) and 14.96 mm (*L. fermentum*) against *S. aureus*, exceeding both the supplementation-only estimates and the prior culture-conditions outcomes by 14-27%.

The two strains therefore respond similarly but not identically to supplementation, and applying the supplement and culture-condition optima together yielded an additive improvement in activity; because the gains were not tested statistically and the fixed NaCl level was not represented in the supplement design, this combination is best described as additive rather than as a demonstrated synergy. Importantly, the models were only marginally significant overall and showed a significant lack-of-fit with a predicted R² of 0.00%; the identified parameters therefore represent a preferable concentration range (an interval estimate) rather than an accurate global optimum (a point estimate), and cannot on their own support quantitative prediction. For this reason, follow-up work should adopt more robust optimization frameworks - for example response transformation, artificial neural networks or genetic-algorithm-assisted optimization - or an expanded multi-factor design that jointly includes NaCl, temperature and pH, together with multi-point, triplicate validation. Within these clearly stated limitations, the optimized, fully food-grade medium provides a promising preliminary basis for the continued development of *L. lactis* MT186647 and *L. fermentum* MT186598 as bacteriocin-producing starter or protective cultures for *P. macrophylla* and related fermented foods. A cross-validated re-analysis with machine-learning regressors (Section 3.6) confirmed that the second-order models are not predictive (negative Q²) and that, although flexible models recover a modest predictive signal (Q² ≈ 0.6), a robust optimum will require an augmented, triplicate design; Gaussian-process or ensemble models fitted to that expanded dataset are the recommended framework for definitive optimization.

## Data Availability

The original contributions presented in the study are included in the article/**Supplementary Material**. Further inquiries can be directed to the corresponding authors.

## References

[B1] Abbasiliasi S. Tan J. S. Tengku Ibrahim T. A. Bashokouh F. Ramakrishnan N. R. Mustafa S. . (2017). Fermentation factors influencing the production of bacteriocins by lactic acid bacteria: a review. RSC. Adv. 7, 29395–29420. doi: 10.1039/C6RA24579J

[B2] Bogovic Matijašić B. Rogelj I. Batič M. Raspor P. (2001). Influence of pH on bacteriocin production by Lactobacillus K7 during batch fermentation. Period. Biol. 103, 163–167.

[B3] Carr F. J. Chill D. Maida N. (2002). The lactic acid bacteria: a literature survey. Crit. Rev. Microbiol. 28, 281–370. doi: 10.1080/1040-840291046759 12546196

[B4] Castro M. P. Palavecino N. Z. Herman C. Garro O. A. Campos C. A. (2011). Lactic acid bacteria isolated from artisanal dry sausages: characterization of antibacterial compounds and study of the factors affecting bacteriocin production. Meat. Sci. 87, 321–329. doi: 10.1016/j.meatsci.2010.11.006 21131135

[B5] Chapot-Chartier M.-P. Kulakauskas S. (2014). Cell wall structure and function in lactic acid bacteria. Microb. Cell Fact. 13, S9. doi: 10.1186/1475-2859-13-S1-S9 25186919 PMC4155827

[B6] Cladera-Olivera F. Caron G. R. Brandelli A. (2004). Bacteriocin production by Bacillus licheniformis strain P40 in cheese whey using response surface methodology. Biochem. Eng. J. 21, 53–58. doi: 10.1016/j.bej.2004.05.002 38826717

[B7] Darbandi A. Asadi A. Mahdizade Ari M. Ohadi E. Talebi M. Halaj Zadeh M. . (2022). Bacteriocins: properties and potential use as antimicrobials. J. Clin. Lab. Anal. 36, e24093. doi: 10.1002/jcla.24093 34851542 PMC8761470

[B8] Dorau R. Liu J. Solem C. Jensen P. R. (2021). “ Metabolic engineering of lactic acid bacteria,” in Metabolic Engineering: Concepts and Applications, (Weinheim, Germany: Wiley-VCH GmbH) 13, 551–610. doi: 10.1002/9783527823468.ch15

[B9] Himelbloom B. Nilsson L. Gram L. (2001). Factors affecting production of an antilisterial bacteriocin by Carnobacterium piscicola strain A9b in laboratory media and model fish systems. J. Appl. Microbiol. 91, 506–513. doi: 10.1046/j.1365-2672.2001.01401.x 11556917

[B10] Hossain T. J. (2022). Functional genomics of the lactic acid bacterium Limosilactobacillus fermentum LAB-1: metabolic, probiotic and biotechnological perspectives. Heliyon 8(11), e11412. doi: 10.1016/j.heliyon.2022.e11412 36387576 PMC9647476

[B11] Ibejekwe A. N. Egbere J. O. Dashen M. M. Ngene A. C. Akpagher S. F. Adetunji J. A. . (2023). Preliminary investigations on the therapeutic efficacy and safety of mixed probiotic lactic acid bacteria on albino rats challenged with Shigella dysenteriae. Adv. Gut. Microb. Res. 2023, 1917108. doi: 10.1155/2023/1917108

[B12] Ibejekwe A. N. Egbere J. O. Dashen M. M. Ngene A. C. McNeil R. T. (2025). Modulatory effects of Lactococcus lactis on gut microbiota of albino rats induced with Escherichia coli O175:H7. Microbes Infect. Dis. 6, 6743–6752. doi: 10.21608/mid.2024.276664.1841

[B13] John O. P. Afolabi K. O. Ngene A. C. Tanimowo W. O. Adewoyin M. A. Osho M. B. . (2026). Enterococcus species: multifaceted probiotic potential and safety considerations. Microorganisms 14, 815. doi: 10.3390/microorganisms14040815 42075212 PMC13118434

[B14] Juarez Tomás M. S. Bru E. Wiese B. De Ruiz Holgado A. A. P. Nader-Macías M. E. (2002). Influence of pH, temperature and culture media on the growth and bacteriocin production by vaginal Lactobacillus salivarius CRL 1328. J. Appl. Microbiol. 93, 714–724. doi: 10.1046/j.1365-2672.2002.01753.x 12234356

[B15] Kaur B. Garg N. Sachdev A. (2013). Optimization of bacteriocin production in Pediococcus acidilactici BA28 using response surface methodology. Asian J. Pharm. Clin. Res. 6, 192–195.

[B16] Kumar M. Jain A. K. Ghosh M. Ganguli A. (2012). Statistical optimization of physical parameters for enhanced bacteriocin production by L. casei. Biotechnol. Bioprocess. Eng. 17, 606–616. doi: 10.1007/s12257-011-0631-4 30311153

[B17] Le N. T. T. Bach L. G. Nguyen D. C. Le T. H. X. Pham K. H. Nguyen D. H. . (2019). Evaluation of factors affecting antimicrobial activity of bacteriocin from Lactobacillus plantarum microencapsulated in alginate-gelatin capsules and its application on pork meat as a bio-preservative. Int. J. Environ. Res. Public Health 16, 1017. doi: 10.3390/ijerph16061017 30897806 PMC6466082

[B18] Lee Y. M. Kim J. S. Kim W. J. (2012). Optimization for the maximum bacteriocin production of Lactobacillus brevis DF01 using response surface methodology. Food Sci. Biotechnol. 21, 653–659. doi: 10.1007/s10068-012-0085-2 30311153

[B19] Musa S. Egbere J. O. Odu C. E. Danladi M. M. Onyimba I. A. Dashen M. . (2023). In-vitro inhibitory effect of lactic acid bacteria isolated from Kunun zaki against multi-drug resistant diarrhogenic bacteria in HIV patients in Jos, Nigeria. Microbes Infect. Dis. 4. doi: 10.21608/mid.2023.175533.1418

[B20] Ngene A. C. Azi H. Y. Dashen M. M. Egbere O. J. (2026). Indigenous probiotics in African poultry production: potentials, mechanisms, and policy directions for combating antimicrobial resistance. World's. Poultry. Sci. J., 1–46. doi: 10.1080/00439339.2026.2652095 37339054

[B21] Ohaegbu C. G. Ngene A. C. Asuquo U. I. Coulthard O. D. Nwachukwu E. (2022). Characterization and antimicrobial activities of lactic acid bacteria isolated from selected Nigerian traditional fermented foods. Afr. J. Biotechnol. 21, 218–236. doi: 10.5897/AJB2021.17450

[B22] Onwuakor C. E. Nwaugo V. O. Nnadi C. J. Emetole J. M. (2014). Effect of varied culture conditions on crude supernatant (bacteriocin) production from four Lactobacillus species isolated from locally fermented maize (Ogi). Am. J. Microbiol. Res. 2, 125–130. doi: 10.12691/ajmr-2-5-1

[B23] Onwuakor C. E. Ogbulie J. N. Braide W. Nwokafor C. V. Uchendu C. E. (2021). Optimization of bacteriocin production by Lactobacillus fermentum strain COE20 from fermenting Pentaclethra macrophylla Benth using response surface methodology. Am. J. Food. Sci. Technol. 9, 30–37. doi: 10.12691/ajfst-9-2-1

[B24] Onwuakor C. E. Ogbulie J. N. Braide W. Ogbulie T. E. Nwokafor C. V. Uchendu C. E. (2020). Optimization of culture conditions for enhanced bacteriocin production by Lactococcus lactis MT186647 using response surface methodology. Am. J. Microbiol. Res. 8, 110–116. doi: 10.12691/ajmr-8-4-1

[B25] Radha K. R. Padmavathi T. (2017). Statistical optimization of bacteriocin produced from Lactobacillus delbrueckii subsp. bulgaricus isolated from yoghurt. Int. Food. Res. J. 24, 803–809.

[B26] Sarabia L. A. Ortiz M. C. (2009). “ Response surface methodology,” in Comprehensive Chemometrics, vol. 1 . Eds. Brown S. Tauler R. Walczak B. ( Elsevier), 345–390.

[B27] Sidooski T. Brandelli A. Bertoli S. L. Souza C. K. D. Carvalho L. F. D. (2019). Physical and nutritional conditions for optimized production of bacteriocins by lactic acid bacteria - a review. Crit. Rev. Food Sci. Nutr. 59, 2839–2849. doi: 10.1080/10408398.2018.1474852 29746783

[B28] Suganthi V. Mohanasrinivasan V. (2015). Optimization studies for enhanced bacteriocin production by Pediococcus pentosaceus KC692718 using response surface methodology. J. Food Sci. Technol. 52, 3773–3783. doi: 10.1007/s13197-014-1440-5 26028762 PMC4444917

[B29] Tian S. Ma S. Xia Y. Huang K. (2026). Lactic acid bacteria bacteriocins: classification, biosynthesis, health benefits, and strategies for enhanced efficacy. J. Agric. Food. Chem. 74, 5859–5871. doi: 10.1021/acs.jafc.5c14047 41700423

[B30] Vázquez J. A. Cabo M. L. González M. P. Murado M. A. (2004). The role of amino acids in nisin and pediocin production by two lactic acid bacteria: a factorial study. Enzyme Microb. Technol. 34, 319–325. doi: 10.1016/j.enzmictec.2003.11.005 38826717

[B31] Yolmeh M. Jafari S. M. (2017). Applications of response surface methodology in the food industry processes. Food Bioprocess. Technol. 10, 413–433. doi: 10.1007/s11947-016-1855-2 30311153

[B32] Zhang Y. Li L. Pang X. Zhang S. Liu Y. Wang Y. . (2026). Lactic acid bacteria as the green and safe food preservatives: their mechanisms, applications and prospects. Foods 15, 241. doi: 10.3390/foods15020241 41596840 PMC12839946

