# Supplementary material for: Statistical optimization of glucose, sodium citrate and potassium chloride supplementation for enhanced bacteriocin production by Lactococcus lactis MT186647 and Lactobacillus fermentum MT186598 isolated from fermenting Pentaclethra macrophylla benth
Source: Front Antibiot. 2026 Jul 22;5:1907088. doi: 10.3389/frabi.2026.1907088 (PMC13445716; doi:10.3389/frabi.2026.1907088)
Supplement: Supplementary file 1 [file SupplementaryFile1.docx]

Supplementary Material

**Statistical optimization of glucose, sodium citrate and potassium chloride supplementation for enhanced bacteriocin production by Lactococcus lactis MT186647 and Lactobacillus fermentum MT186598 isolated from fermenting Pentaclethra macrophylla Benth.**

*Chijioke Ethel Onwuakor and Anayochukwu Chibuike Ngene*

# Contents of the supplementary material

- Supplementary Data S1: Central composite design dataset used for regression modelling and predictive validation.
- Supplementary Method S1: Non-parametric re-analysis and leave-one-out cross-validation workflow.
- Supplementary Table S1: Predictive performance of the fitted models.
- Supplementary Table S2: Optima located by grid search over the experimental design region.

# Supplementary Data S1

The dataset comprises the 20 runs of the central composite design. Factor levels are uncoded. Response values are the mean zones of inhibition from duplicate fermentations against Staphylococcus aureus ATCC 19095. The coefficient of variation between duplicates did not exceed 4.5% in any run. Individual duplicate observations were not reported in the article and are therefore not reconstructed here.

**Supplementary Data S1.** Central composite design matrix and observed bacteriocin activities used for model fitting and predictive validation.

| **Run** | **Glucose (g/L)** | **Sodium citrate (g/L)** | **KCl (g/L)** | **L. lactis MT186647 ZI (mm)** | **L. fermentum MT186598 ZI (mm)** |
| --- | --- | --- | --- | --- | --- |
| 1 | 5 | 0.3 | 0.55 | 10.22 | 9.85 |
| 2 | 10 | 0.3 | 0.55 | 9.35 | 8.92 |
| 3 | 5 | 1.0 | 0.55 | 9.23 | 9.66 |
| 4 | 10 | 1.0 | 0.55 | 9.44 | 8.12 |
| 5 | 5 | 0.3 | 3.0 | 10.85 | 10.20 |
| 6 | 10 | 0.3 | 3.0 | 9.88 | 9.02 |
| 7 | 5 | 1.0 | 3.0 | 9.87 | 9.12 |
| 8 | 10 | 1.0 | 3.0 | 9.28 | 8.87 |
| 9 | 3.3 | 0.65 | 1.78 | 13.62 | 12.89 |
| 10 | 11.7 | 0.65 | 1.78 | 9.12 | 8.65 |
| 11 | 7.5 | 0.06 | 1.78 | 12.21 | 12.33 |
| 12 | 7.5 | 1.24 | 1.78 | 10.14 | 10.22 |
| 13 | 7.5 | 0.65 | 1.78 | 12.55 | 12.11 |
| 14 | 7.5 | 0.65 | 3.84 | 10.98 | 10.21 |
| 15 | 7.5 | 0.65 | 1.78 | 12.85 | 12.22 |
| 16 | 7.5 | 0.65 | 1.78 | 12.83 | 12.58 |
| 17 | 7.5 | 0.65 | 1.78 | 13.21 | 12.56 |
| 18 | 7.5 | 0.65 | 1.78 | 12.71 | 12.52 |
| 19 | 7.5 | 0.65 | 1.78 | 12.98 | 12.35 |
| 20 | 7.5 | 0.65 | 1.78 | 12.54 | 12.40 |

# Supplementary Method S1

## Non-parametric re-analysis and predictive validation

The 20-run dataset for each strain was analysed independently. Five non-parametric or machine-learning regressors were examined alongside an ordinary least-squares second-order polynomial baseline: Gaussian-process regression with a radial-basis-function kernel and white-noise term, support-vector regression with a radial-basis-function kernel, k-nearest-neighbour regression with k = 3, random-forest regression, and gradient-boosted-tree regression.

Predictive performance was evaluated by leave-one-out cross-validation. In each fold, one experimental run was withheld, the model was fitted to the remaining 19 runs, and the fitted model was used to predict the withheld response. Predictor standardisation was performed within each cross-validation fold to prevent information leakage. The predictions from all 20 folds were pooled to calculate the cross-validated coefficient of determination (Q²). The manuscript also specifies root-mean-square error and mean absolute error as evaluation measures, although their numerical values are not reported in the article proof.

The optimum of each fitted response surface was located by exhaustive search over a dense grid spanning the experimental design region. Optima occurring on a sampled boundary were treated as unresolved boundary solutions rather than confirmed global optima. Analyses were performed in Python 3.12 using scikit-learn 1.8.

# Supplementary Table S1

**Supplementary Table S1.** Leave-one-out cross-validated predictive performance of the fitted models.

| **Model** | **R² (in) L. lactis** | **Q² (LOOCV) L. lactis** | **R² (in) L. fermentum** | **Q² (LOOCV) L. fermentum** |
| --- | --- | --- | --- | --- |
| Second-order polynomial (baseline) | 0.842 | -0.508 | 0.859 | -0.377 |
| Gaussian process (RBF + noise) | 0.993 | 0.315 | 0.996 | 0.452 |
| Support-vector regression (RBF) | 0.990 | 0.331 | 0.994 | 0.296 |
| k-nearest neighbours (k = 3) | 0.992 | 0.078 | 0.995 | 0.027 |
| Random forest | 0.938 | 0.521 | 0.951 | 0.625 |
| Gradient boosting | 0.989 | 0.631 | 0.987 | 0.602 |

**R² (in)** is the in-sample coefficient of determination. **Q² (LOOCV)** is the leave-one-out cross-validated predictive coefficient of determination. A negative Q² indicates no out-of-sample predictive power relative to prediction by the overall mean.

# Supplementary Table S2

**Supplementary Table S2.** Optima located by grid search over the experimental design region for each model.

| **Model** | **L. lactis optimum (glucose/citrate/KCl g/L -> mm)** | **L. fermentum optimum (glucose/citrate/KCl g/L -> mm)** |
| --- | --- | --- |
| Second-order polynomial (original) | 6.35/0.55/1.78 -> 13.12 | 6.44/0.55/1.59 -> 12.74 |
| Gaussian process | 5.19/0.64/0.55 -> 14.21 | 7.41/0.42/0.55 -> 13.17 |
| Support-vector regression | 4.16/0.59/1.83 -> 13.61 | 5.70/0.57/1.83 -> 13.06 |
| Random forest | 3.30/0.49/1.83 -> 13.05 | 6.39/0.49/1.22 -> 12.39 |
| Gradient boosting | 3.30/0.49/1.22 -> 13.60 | 3.30/0.49/1.22 -> 12.87 |

Boundary interpretation: glucose = 3.30 g/L is the lower axial level. Potassium chloride = 0.55 g/L is the lower sampled bound; the design did not contain a low axial point for potassium chloride. An optimum located at either boundary represents extrapolation towards the edge of the sampled region rather than a resolved interior optimum.
